# Active Learning by Design: An Undergraduate Introductory Public Health Course

**DOI:** 10.3389/fpubh.2014.00284

**Published:** 2014-12-23

**Authors:** Karin B. Yeatts

**Affiliations:** ^1^Department of Epidemiology, Gillings School of Global Public Health, University of North Carolina at Chapel Hill, Chapel Hill, NC, USA

**Keywords:** undergraduate, public health, introductory, active, learning

## Abstract

Principles of active learning were used to design and implement an introductory public health course. Students were introduced to the breadth and practice of public health through team and individual-based activities. Team assignments covered topics in epidemiology, biostatistics, health behavior, nutrition, maternal and child health, environment, and health policy. Students developed an appreciation of the population perspective through an “experience” trip and related intervention project in a public health area of their choice. Students experienced several key critical component elements of a public health undergraduate major; they explored key public health domains, experience public health practice, and integrated concepts with their assignments. In this paper, course assignments, lessons learned, and student successes are described. Given the increased growth in the undergraduate public health major, these active learning assignments may be of interest to undergraduate public health programs at both liberal arts colleges and research universities.

## Introduction

### Growing interest in the undergraduate public health major

A recent report by Leider et al. (1) shows a dramatic increase (750%) in undergraduate degree public health conferrals from 759 in 1992 to 6,464 in 2012. With the growth in the undergraduate public health major, there will be an increase in demand for introductory public health courses. In this paper, an introductory public health course designed using active learning principles and Liberal Education and America’s Promise (LEAP) outcomes (2, 3) is described. The assignments may be of interest to a range of institutions offering or planning to offer an undergraduate public health major.

### Undergraduate public health critical core components

In 2003, the IOM called for incorporating public health into undergraduate education. Since that time, AAPH and Association of Liberal Arts Colleges developed critical component elements (CCEs) of a public health education and the Association of American Colleges and Universities LEAP outcomes (2, 4–6). LEAP learning outcomes (2, 5, 6) include intellectual and practical skills such as inquiry and analysis, critical and creative thinking, written and oral communication, quantitative literacy, information literacy, teamwork, and problem solving. The CCEs include a broad education with liberal arts, a breadth of understanding of public health domains, and experiential knowledge of the field (4). LEAP principles and the CCEs both encompass personal and social responsibility as well as integrative learning (4, 6).

More than 20 years ago, Barr and Tagg (7) elegantly described the shift from an “Instruction Paradigm” to a “Learning Paradigm” with respect to learning theory. Active learning, is defined as “any instructional method that engages students in the learning process” and is in contrast to students receiving information passively from the instructor (8). Reviews of the literature on active learning by Michael (9) and Prince (8) make several key points. First, active learning works across disciplines of physics, biology, and chemistry and engineering (8, 9). Most recently in 2014, Freeman (10) showed that students learned better in an active learning environment compared with a passive lecture format in engineering, mathematics, and natural sciences (10).

One facet of active learning is collaborative team-based learning “individuals are more likely to learn more when they learn with others than when they learn alone” (9). Collaborative efforts fit well with Fink’s detailed descriptions of creating significant learning experiences (11). Team learning in pharmacy (12) and nursing (13) has shown increased levels of engagement and enhanced critical thinking. While literature in the public health field is more limited, Kjellgren (14) and Goldman (15) provide evidence that active learning techniques such as reflective journals and blogging led to students increased questioning of their own understanding and reporting of enriched learning on course topics.

### Integrating active learning with course objectives

In this course, students are provided with multiple opportunities to apply and integrate concepts. The course objectives listed in Table 1 were created in consideration of current undergraduate public health domains and LEAP learning outcomes (2, 4, 5, 16). These domains and learning outcomes include inquiry and analysis, critical and creative thinking, written and oral communication, quantitative literacy, information literacy, teamwork, and problem solving (2–6, 16).

**Table 1:** **List of course objectives**.

A. Recognize local to global aspects of public health and appreciate the field’s breadth and core values
B. Describe key events in the history of public health and their influence on the development of today’s public health approaches
D. Identify and describe major health-related needs of populations using evidence-based data
C. Identify the determinants of health (socioeconomic, behavioral, biological, environmental, etc.) that affect health of individuals and populations across the life course
F. Critically evaluate public health interventions for evidence for relevance, application, and evaluation
G. Describe how the characteristics and organizational structures (including health policies, regulations, ethics, and economics) of health systems contribute to public health issues in the US and other countries
H. Value the ethical considerations in human subject research
I. Effectively communicate public health concepts

### Target audience

Our target audience for this course is composed of undergraduate honors students ranging from freshmen to seniors. Students from all majors are welcome to take the course. In the first 3 years, the diverse group of majors ranged from biology and chemistry to anthropology, business, and English. In terms of course recruitment, information on the course offering is circulated each summer by the Honor’s Program Associate Director of Curriculum. In the future, we would like to open the course to all undergraduate students.

### Course structure and content

#### Overview

The course is structured with two major components. The first component comprises a series of team-based activities; the second component comprises an experience trip and intervention project. In Table 2, we illustrate how team and individual assignments align with various course objectives. The first course component incorporates team activities that explore various public health disciplines in greater depth. In the second course component, students select and visit a local public health organization, and then use their experience to inform their final project examining a public health intervention. The activities for each of the two components are described in more detail below.

**Table 2:** **Class assignment alignment with course objectives**.

Activity	Type		Course objectives addressed
	Team	Individual	
Population perspective: students use Hans Rosling’s Gapminder, http://www.gapminder.org/, to investigate at a global level how population health varies by place, time, and macro-level factors such as a country’s Gross Domestic Product (GDP) or health care accessibility	x		A. Recognize local to global aspects of public health and appreciate the field’s breadth and core values
			B. Describe key events in the history of public health and their influence on the development of today’s public health approaches
			D. Identify and describe major health-related needs of populations using evidence-based data
Supertasters: students are introduced to concepts and tools of human health data collection	x		D. Identify and describe major health-related needs of populations using evidence-based data
			H. Value the ethical considerations in human subject research
			I. Effectively communicate public health concepts
Market basket assignment: students are given a “basket of food” that they price at various food outlets to compare the cost of eating healthy vs. un-healthy		x	C. Identify the determinants of health (socioeconomic, behavioral, biological, environmental, etc.) that affect health of individuals and populations across the life course
			D. Identify and describe major health-related needs of populations using evidence-based data
Social media campaign: each team selects a health behavior (such as hand washing) or an organization that promotes healthy behavior. Teams then define a target population and formulate a specific message for their population. Next, teams create a social media video with the message to encourage/support a behavioral change	x		A. Recognize local to global aspects of public health and appreciate the field’s breadth and core values
			C. Identify the determinants of health (socioeconomic, behavioral, biological, environmental, etc.) that affect health of individuals and populations across the life course
			I. Effectively communicate public health concepts
Clean or contaminated water: students learn about environmental health through environmental water sampling and mapping	x		A. Recognize local to global aspects of public health and appreciate the field’s breadth and core values
			C. Identify the determinants of health (socioeconomic, behavioral, biological, environmental, etc.) that affect health of individuals and populations across the life course
			I. Effectively communicate public health concepts
Health care race: students gain personal experience with the user end of the health care system	x		C. Identify the determinants of health (socioeconomic, behavioral, biological, environmental, etc.) that affect health of individuals and populations across the life course
			G. Describe how the characteristics and organizational structures (including health policies, regulations, ethics and economics) of health systems contribute to public health issues in the US and other countries
			H. Value the ethical considerations in human subject research
			I. Effectively communicate public health concepts
Ecosystem of health care: students are asked to draw a diagram with interactions among various entities in the health care system, including patients, insurers, and industry	x		G. Describe how the characteristics and organizational structures (including health policies, regulations, ethics, and economics) of health systems contribute to public health issues in the US and other countries
Experience trip reflection: students engage in a public health activity outside of the classroom and reflect the goal of the program or activity, what they observed and how it fits with public health concepts learned in class		x	A. Recognize local to global aspects of public health and appreciate the field’s breadth and core values
			C. Identify the determinants of health (socioeconomic, behavioral, biological, environmental, etc.) that affect health of individuals and populations across the life course
			F. Critically evaluate public health interventions for evidence for relevance, application, and evaluation
Final project. students examine or propose an intervention related to their experience trip topic		x	A. Recognize local to global aspects of public health and appreciate the field’s breadth and core values
			C. Identify the determinants of health (socioeconomic, behavioral, biological, environmental, etc.) that affect health of individuals and populations across the life course
			F. Critically evaluate public health interventions for evidence for relevance, application, and evaluation
			I. Effectively communicate public health concepts

#### Component 1-team activities

Throughout the semester, students engage in a series of team assignments that allow them to actively learn about the various public health disciplines. Students are introduced to a wide range of different disciplines and topics within the field of public health-including environmental science, nutrition, health behavior, maternal and child health, biostatistics, epidemiology, and health policy. As the course progresses, students discuss protecting the public’s health from different aspects including behavioral education, environment, and policy.

##### Population perspective

Students use Hans Rosling’s Gapminder, http://www.gapminder.org/, to investigate at a global level how population health varies by place, time, and macro-level factors such as a country’s Gross Domestic Product (GDP) or health care accessibility. This tool helps to clarify the ecologic study design and its contribution to generating hypotheses. With the free online data visualization tool, we explain the population perspective and contrast this with the biomedical perspective of health. Students are able to link health outcomes such as infant mortality with historic events such as droughts, famine, or war. For example, we discuss the Great Famine (1958–1961) in China and the large increase in infant and total mortality during this time period.

##### Supertasters

Students are introduced to concepts and tools of human health data collection. The super taster activity is designed to engage students in epidemiological concepts of measurement and screening test sensitivity and specificity. Students compare the sensitivity and specificity of two different tests with a “gold standard” for identifying “Supertasters,” individuals who “experience the five basic tastes with greater intensity” (http://www.bbc.co.uk/science/0/22941835). The first test, a strip of propylthiouracil (PROP), is our “gold standard” because of its ease of use and apparent objectivity. The second test is a short questionnaire; the third test is a count of the number of papillae on the tongue in 1 cm area. In teams, students collect data from their own team members, and then build a dataset for the entire class. Students are introduced to human subject research concepts of confidentiality, and deductive disclosure in a discussion of the privacy of their own test results. Students use the data set to calculate sensitivity and specificity of two tests compared to the gold standard.

##### Social media campaign

Each team selects a health behavior (such as hand washing) or an organization that promotes healthy behavior. Teams then define a target population and formulate a specific message for their population. Next, teams create a social media video with the message to encourage/support a behavioral change. Using digital media, each team produces a video and publishes the campaign online. For example, a student team chose proper hand washing technique ^1^, targeting the student population at UNC Chapel Hill. Another student team promoted the use of a campus organization “Safe Walk,” which provides safety to students walking home late at night.

##### Clean or contaminated water?

Students learn about environmental health through environmental water sampling. Each team selects and defines a different water sampling location (bathroom in dorm, kitchen in apartment, office, airport bathrooms, local creek, University Lake, etc.). Each team receives a low cost water testing kit; some teams test microbial contamination, other teams test chemical contamination. Students also identify their local watershed and drinking water source. Results are presented in class and students discuss variations in sampling sites and results.

##### Health care race

Students gain personal experience with the user end of the health care system. Teams are assigned a specific rural county and a description of a person with a health issue as well as financial/access restrictions. Teams identify local medical providers in the county, and record how long it would take appointment with a health care provider. They also search for transportation options if the person did not have their own transportation. Students answer the question “In a rural area, how long would it take for an elderly person with a pressing health issue to see doctor? “ Each team reports on both successes and challenges.

##### Ecosystem of health care

Students are asked to draw a diagram with interactions among various entities in the health care system, including patients, insurers, and industry. They reflect upon how the Affordable Care Act is starting to change the medical care system at the state and federal levels.

##### Introduction to local public health related organizations/programs

While the large majority of the classes are with one professor, occasionally speakers come from local organizations involved with public health issues. Organizations range from those that help young mothers (Durham Connects, Horizons) to those that increase healthy food availability to low income families and change organic local food market networks (Farmer Foodshare), to those that help individuals lift themselves out of poverty (Community Empowerment Fund).

#### Component 2 – experience trip and final project

We follow Wykoff et al.’s (4) recommendation that “students should be exposed to local-level public health professionals and/or agencies that engage in population health practice.” This assignment integrates course concepts with public health practice in a two-part assignment. First, students conduct an “experience trip.” Then, for their final project students examine or propose an intervention connected with their experience trip topic.

The experience trip is designed for students to come into contact with public health practice. Students are asked to select a local organization (or with a local branch) or program that either directly or indirectly deals with public health. Students are asked to observe, ask questions, and find out about this organization and its activities. The experience takes about 1–3 h. Students write a reflection on their experience trip and answer questions on the goals of the organization, why they chose this topic, their observations, how it fits with public health concepts learned in class, and what they learned from the experience.

We would like students to have a “direct encounter with the phenomena being studied rather than merely thinking about the encounter, or only considering the possibility of doing something about it” (17, 18). By visiting an organization that deals with a public health issue, students have the opportunity to meet individuals that are engaged in giving or receiving some type of intervention and see the environment in which the intervention is conducted. Thus, students can be introduced to a “complex problem in a complex setting, rather than simplified problems in isolation” (17). Their reflection encourages students to synthesize their practical experience with theoretical knowledge.

For the final project paper, students examine an intervention related to their experience trip topic. For example, some students visited local organizations, which deal with increasing nutritional food availability to impoverished primary schoolchildren. Then, for their final project, the students examined existing interventions dealing with food insecurity. Students review the literature related to the intervention, and outline the factors that define the problem the intervention is designed to address. Next, they describe how the intervention was implemented, and explain relevant policies, regulations, ethics, and economics that influence the selected problem and intervention being explored. Lastly, they communicate their analysis in the form of a written report and an in class presentation to their classmates.

## Results and Discussion

### Increasing interest in public health

The UNC Gillings School of Global Public Health has four strong undergraduate BSPH programs – with approximately 215 students enrolled in biostatistics, environmental health, health policy and management, and nutrition. However, with a total of approximately 18,350 UNC undergraduates, there are many UNC undergraduates who have no exposure to the public health field or the School. This course aimed to begin addressing that lack of exposure. In the first 2 years of the course offering, on average 27% of the students who took the introductory public health course applied to the UNC BSPH programs. Of the students who applied, 90% were accepted.

Student feedback on the course demonstrated increasing student excitement and interest in public health. For example, students expressed their appreciation of the experience trip/final project assignment:

“[the professor] gave us the freedom to choose any local organization that addresses a public health issue, and she provided plenty of ideas and resources for us to get started. This final project allowed me to learn about TABLE and the issue of local childhood hunger, which I am now passionate about.” “As my cumulative project, I got to research food deserts and food insecurity, and learned that this is something I am very passionate about.”

### Lessons learned

#### Understanding the population perspective

One lesson learned from teaching the course in the first 2 years was the need to foster and encourage students to approach their experience trip and public health topics from the a population perspective. For example, attending an Alcoholics Anonymous meeting, several students struggled with stepping out of a biomedical perspective; they only saw the individual’s addiction being the result of their own actions, and were initially not able to recognize additional social–ecological influences on patterns of behavior. For these students, the biomedical perspective was entrenched. With faculty encouragement, the students worked hard to understand the population perspective.

Sometimes students approach community agencies, organizations, and self-help groups without an adequate understanding of the population that the organization serves. For instance, AA and NA are designed for recovering alcoholics and addicts; these are social recovery programs, and provide social support in obtaining and maintaining sobriety. These programs do not aspire to be medical model approaches. Students often miss this subtlety. Likewise, a community garden intended to serve an immigrant population would want to plant crops that appeal to their consumer population, rather than follow dietary principles taught in nutrition class. Students need to learn to articulate a client view, rather than a provider view, of the organization and service.

#### Student creativity

One strength of the course, has been the student’s creativity with the focal “experience trip” and final project assignment. Students demonstrated creativity with regards to the selection of an organization or program for their experience trip. For example, one student delved into the problems of youth violence and programs that address issues of school suspensions and restorative justice. Her interest led her to a local organization Boomerang ^2^ which focuses on teaching resiliency skills to suspended middle and high school students. She began volunteering with the organization, and subsequently took a position working with the organization the next semester.

Another student used a fall break service-learning trip to a migrant farm camp to investigate migrant worker health issues. The problem she said, after she returned, was which problem to select from all the issues facing the migrant worker community. She chose to focus on pesticide exposures among farm workers and their families; she described a lay health advisor education program targeted to both the parents and children to help reduce pesticide exposures. Allowing the students to pick an organization or program that fits their interest gives them the opportunity to explore new topics and integrate public health issues in ways that substantially extend course learning. As noted by Cashman (17), public health practice lends itself to experiential learning.

#### Reflections on achieving LEAP learning outcomes

Graded team and individual projects throughout the semester provide evidence of students achieving LEAP outcomes. As described above, the completed assignments illustrated systematic inquiry and analysis on a variety of public health topics. Critical and creative thinking was evident on the team assignments, as well as on the student experience trip reflections and final projects. Students practiced written and oral communication throughout the semester in their team assignments and class presentations. Students also worked on problem solving and developing information literacy in their assignments. Next steps will include a more formal evaluation of changes in LEAP outcomes of intellectual and practical skills.

#### Scalability/community capacity

This class was designed as honors seminar with a class size of 24 students. With the current course structure, there are some limitations of scalability. Some institutions have large programs with hundreds of students interested in an introduction to public health. The course presented here, with some reorganization and redesign, could be scaled up. Community capacity needs to be considered. In order for each student to successfully complete their project, there must be enough community resources for all to have an experience trip without overburdening community resources. This may be more challenging for schools that are in geographically isolated areas. However, in 2012 the majority (73%) of students receiving an undergraduate public health degree lived in cities (1).

It is possible to increase the number of visitation sites in several ways: for example, there are often multiple AA and NA meetings throughout the week. There may be local agencies with multiple programs, and there may be sites, such as a community garden, that can accommodate any number of students. Despite this challenge, in the future, we would like to incorporate a larger service-learning component.

Given the increasing rise of the undergraduate public health major, these active learning assignments may be of interest to undergraduate public health programs at institutions ranging from liberal arts colleges to research universities. The advantage of a broad introductory course like this one is providing the next generation of students with basic public health literacy. Additional benefits include capturing student interest early on in their professional development, and introducing them to potential previously unrecognized career options. Yet, while only a small proportion of students may become public health professionals, the course promotes the values of protecting the public’s health to all students. The ultimate benefit is increased awareness and support of public health in our global society.

## Conflict of Interest Statement

The author declares that the research was conducted in the absence of any commercial or financial relationships that could be construed as a potential conflict of interest.

## Footnotes

^1^https://www.youtube.com/watch?v=oRshGys7AUs

^2^http://www.boomerangymca.org/our-program/mission-vision/
